# A Case of Refractory Gastric Cardia Ulcer in Which Marked Elevation of the Surrounding Mucosa was Observed During the Clinical Course

**DOI:** 10.4021/gr546w

**Published:** 2013-07-14

**Authors:** Tateki Yamane, Akira Umeda, Hitoshi Shimao, Takayuki Ishii

**Affiliations:** a Division of Gastroenterology, Department of Internal Medicine, The International University of Health and Welfare, Shioya Hospital, Japan; b Division of Pulmonology, Department Of Internal Medicine, The International University of Health and Welfare, Shioya Hospital, Japan; c Department of Surgery, The International University of Health and Welfare, Shioya Hospital, Japan; d Sanikukai Family Clinic, Japan

**Keywords:** Refractory peptic ulcer, High located gastric ulcer, Recurrence after Hp eradication

## Abstract

A 55-year-old man visited our department because of epigastric pain. Upper gastrointestinal endoscopy revealed a small, undermined ulcer in the gastric cardia. He had no history of taking NSAIDs, and was positive for *Helicobacter pylori* (Hp) infection. After Hp eradication therapy followed by 8 weeks of proton pump inhibitor (PPI) administration, re-endoscopy showed that the ulcer had slightly shrunk without scarring, and the surrounding mucosa was markedly elevated, like in a submucosal tumor. Endoscopic ultrasonography, performed at the same time, showed thickening of the submucosal and muscular layers around the ulcer. After continuous PPI administration, the mucosal elevation disappeared, and the ulcer shrunk and later scarred. However, when the dose of PPI was reduced with the aim of discontinuing it after the confirmation of successful Hp eradication, the ulcer recurred. We report this case of gastric ulcer because of its peculiar clinical presentation.

## Introduction

The development of proton pump inhibitors (PPIs) and introduction of *Helicobacter pylori* (Hp) eradication has made peptic ulcer treatment straightforward and reliable. However, cases (although small in number) of refractory or unusual gastric ulcers remain. We report a case of gastric cardia ulcer in which marked elevation of the surrounding mucosa was observed during the clinical course, which was resistant to PPI therapy, recurring after PPI dose reduction following Hp eradication.

## Case Report

The patient was a 55-year-old man with a history of hypertension who was taking amlodipine. He had no history of taking NSAIDs. He was a social drinker and had no history of smoking. He visited our department because of epigastric pain of 3 months’ duration.

Physical examination revealed no abnormalities other than epigastric tenderness. Laboratory tests showed no abnormalities in the blood cell count or blood chemistry. He was positive for the anti-Hp antibody, and had a normal serum gastrin level.

**Figure 1:**
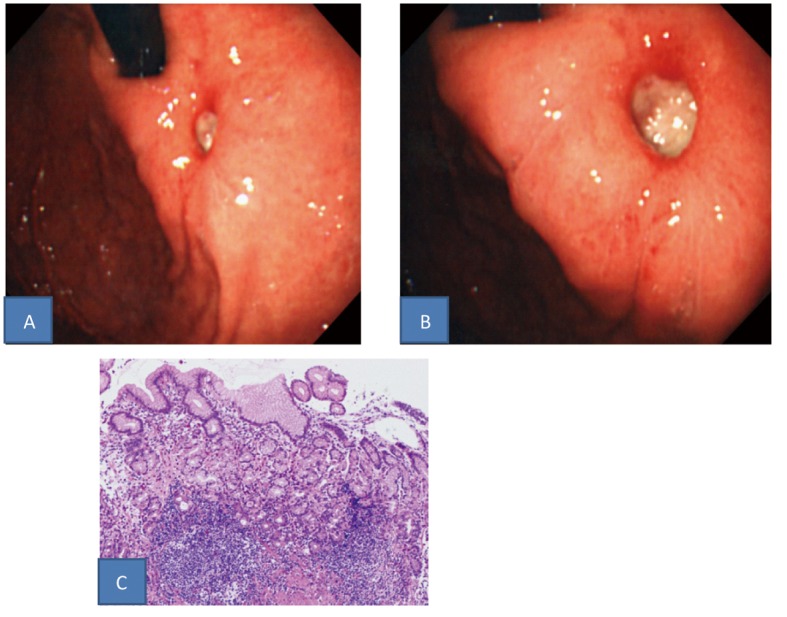
A, B: Upper gastrointestinal endoscopic findings (A: Intermediate view. B: Near view). A small, uedermined ulcer was found in the gastric cardia. C: Histopathological findings in a biopsy specimen (HE stain). Specific findings were not found: only hyperplastic changes of the cryptic epithelium and inflammatory cell infiltration in the storoma.

Upper gastrointestinal endoscopy showed chronic atrophic gastritis and a small (8 mm in long diameter), oval-shaped, undermined ulcer in the anterior wall of the gastric cardia (Fig. 1A, B), but no abnormalities in the esophagus or duodenum.

**Figure 2:**
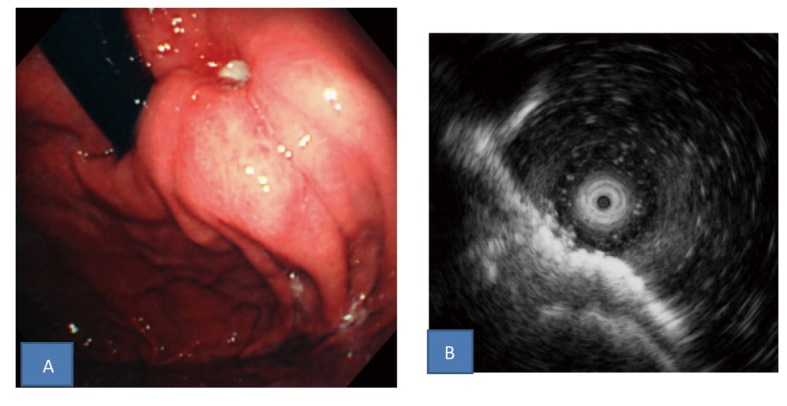
A: Upper gastrointestinal endoscopic findings after Hp eradication therapy followed by 8 weeks of PPI administration. The ulcer slightly shrunk without scarring and the surrounding mucosa was markedly elevated like a submucosal tumor. B: Endoscopic ultrasonography findings. Thickening of the submucosal and muscular layers around the ulcer was found.

**Figure 3:**
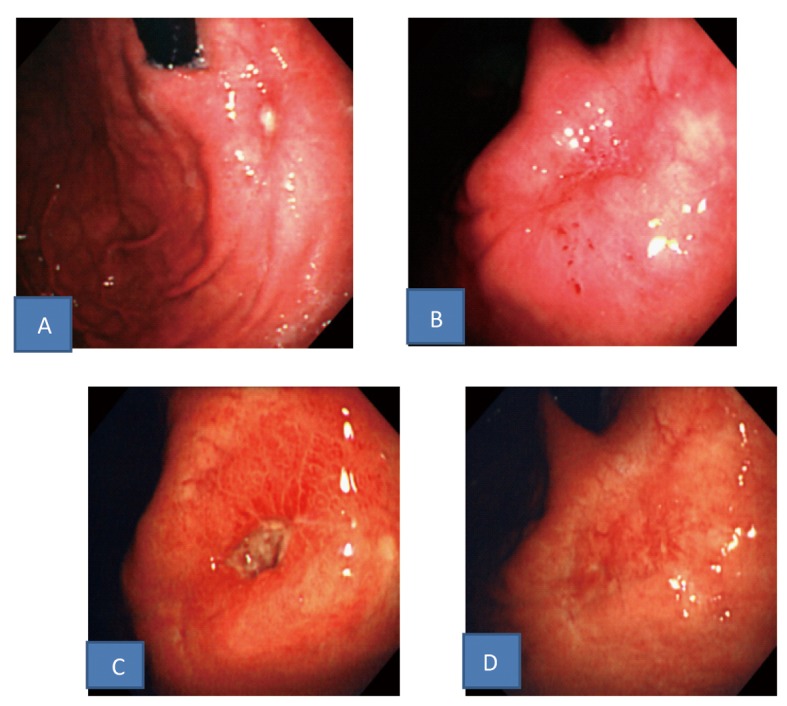
Upper gastrointestinal endoscopic findings. A: The ulcer shrunk and the elevation around the ulcer disappeared after continuous PPI administration. B: The ulcer scarred after more continuous PPI administration. C: The ulcer recurred after PPI dose reduction in spite of successful Hp eradication. D: The ulcer scarred again after increase of PPI dosage.

Histopathological examination of biopsies of the ulcer margin revealed nonspecific findings: only hyperplastic changes of the cryptic epithelium and inflammatory cell infiltration in the stroma (Fig. 1C). After 1 week of triple-drug Hp eradication therapy (lansoprazole (LPZ) 60 mg/day, amoxicillin 1,500 mg/day, and clarithromycin 400 mg/day), followed by 8 weeks of 30 mg/day of LPZ, re-endoscopy showed that the ulcer had slightly shrunk without scarring, and the surrounding mucosa was markedly elevated, like a submucosal tumor (Fig. 2A). Endoscopic ultrasonography, performed at the same time, showed thickening of the submucosal and muscular layers around the ulcer (Fig. 2B). Biopsies were performed again, showing no evidence of malignancy. In addition, abdominal CT revealed no abnormalities outside the gastric wall. Since the symptoms subsided, LPZ (30 mg/day) was continued for 8 weeks, and re-endoscopy was performed, which showed that the ulcer had shrunk, and the elevation around the ulcer had disappeared (Fig. 3A). LPZ administration was continued for 8 more weeks, and then another endoscopy was performed, confirming the cicatrization of the ulcer (Fig. 3B). Based on the anti-Hp antibody profile, Hp eradication was deemed successful. Therefore, LPZ was reduced to 15 mg/day with the aim of tapering and discontinuing it, but epigastric pain recurred, and endoscopy revealed recurrence of the ulcer (Fig. 3C). After the increase of LPZ to 30 mg/day, the symptom subsided, and the ulcer scarred again (Fig. 3D). At present, the patient is taking 30 mg/day of LPZ and is being followed-up.

## Discussion

The cause of this ulcer was considered to be common Hp infection, because the patient had no history of taking any drugs, such as NSAIDs and bisphosphonates, which could cause gastric mucosal damage, and because specific conditions, such as Zollinger-Ellison syndrome and Crohn’s disease, were excluded. This patient had a high located gastric ulcer arising in the gastric cardia. In Japan, gastric cardia ulcer accounts for less than 2% of gastric ulcers [1, 2]. In general, patients with a high located gastric ulcer often present with intestinal metaplasia of the gastric mucosa around the ulcer and suffer from gastric hypoacidity due to the progression of mucosal atrophy, and often have elevated serum gastrin levels, which reflect hypoacidity [2]. However, this case was atypical as high located gastric ulcer in that intestinal metaplasia was not apparent in the gastric mucosa around the ulcer on gross endoscopy or biopsy, and no increase in serum gastrin levels was noted.

In this patient, the ulcer was refractory to PPI, and showed delayed healing. In Japan, PPI-resistant ulcers reportedly account for 2.5-4% of all gastric ulcers [3], and they are morphologically characterized by undermining, linear shape, large size and circumferential elevation [3]. In these cases, histopathologically, there is dense collagen fiber hyperplasia in the ulcer base, and fibrosis is also seen in the mucosa, epithelial regeneration is difficult due to blood flow disturbance, and the epithelium is fragile even if it is regenerated, making ulcer healing difficult [4]. The present ulcer was not large, but was undermined, corresponding to one of the above morphologies of refractory ulcers. In addition, we speculate that, since the ulcer was located in the gastric cardia, it was exposed to abrasive mechanical stimulation by food, thus constituting a contributory factor leading to refractoriness. On the other hand, in patients with a PPI-resistant ulcer, the insufficient action of PPIs resulting from enhanced drug metabolism due to genetic polymorphism and their inactivation in the stomach due to gastric emptying disturbance should be considered [5, 6]. However, it is believed that, during long-term PPI therapy, as in this patient, enhanced drug metabolism due to being a rapid metabolizer does not influence the effect of PPI. Furthermore, in this patient, no stomach deformities, such as wallet stomach, were observed, and no food residues were seen during endoscopy, excluding the possibility of PPI inactivation due to gastric emptying disturbance.

In this patient, Hp eradication was not useful in that PPI dose reduction after Hp eradication resulted in ulcer recurrence. It has been reported that Hp eradication removes mucosa-damaging factors, such as urease and inflammatory cytokines, and is thus useful for improving the healing of refractory gastric ulcers and preventing ulcer recurrence. However, we speculate that, in this patient, the regenerated epithelium was fragile because of blood flow disturbance due to mucosal fibrosis, and Hp eradiation may have adversely affected ulcer healing by increasing gastric acid secretion and thereby strengthening the gastric acid stimulation of the regenerated epithelium.

Further, in this patient, the ulcer-surrounding mucosa was markedly elevated during the clinical course, as in a submucosal tumor, raising concerns about the possibility of a neoplastic lesion, but the mucosal elevation disappeared with the healing of the ulcer. It is reported that such mucosal elevation is rarely seen during the healing of refractory gastric ulcers [4]. This is believed to be due to the delay in the degradation of hyperplastic collagen fibers and their accumulation [4].

Although peptic ulcer treatment has progressed due to PPI therapy and Hp eradication, it should be kept in mind that cases of unusual gastric ulcers, such as that reported here, remain.
